# 

**Published:** 1823-05

**Authors:** 


### MONTHLY CATALOGUE OF MEDICAL BOOKS.

*** *It having been hinted to us that gentlemen, sending copies of their Works, prefer having their titles given at length, it is our intention, hereafter, to comply with this suggestion: but it is to be observed, that no books can be entered on this list except those sent to us for the purpose;—in the lists hitherto transmitted, we find that the names of works have frequently been given as published, which have not appeared for weeks, or even months after.*

Elements of the Theory and Practice of Physic, designed for the use of Students. Part II.; including the Symptoms, Pathology, and Treatment of Chronic Diseases. By GEORGE GREGORY, M.D. Licentiate of the Royal College of Physicians in London, and senior Physician to the St. George's and St. James's Dispensary.—pp. 450. London, 1823.

A Practical Treatise on the Symptoms, Causes, Discrimination, and Treatment, of some of the most important Complaints that affect the Secretion and Excretion of the Urine: the whole exhibiting a comprehensive View of the various Diseases of the Kidneys, Bladder, Prostate Gland, and Urethra. Illustrated by numerous Cases, and Engravings. By JOHN HOWSHIP, Member of the Royal College of Surgeons in London, Societé Medicale d'Emulation of Paris, Royal Medical Society of Edinburgh, and Medico-Chirurgical Society of London, &c. &c. &c.—pp. 438. London, 1823.

A Treatise on Mental Derangement: containing the Substance of the Gulstonian Lectures, for May 1822. By FRANCIS WILLIS, M.D. Fellow of the Royal College of Physicians.—pp. 234. London, 1823.

A Letter to the Right Honourable the Earl of Liverpool, concerning the present State of Vaccination, and pointing out the urgent Necessity of an immediate Investigation, in order to secure the Safety and Comfort of the Public. By THOMAS BROWN, Surgeon, Musselburgh.—*Magna est veritas et prevalebit.*—pp. 43. Edinburgh, 1823.

Thoughts on the present Character and Constitution of the Medical Profession. By T. C. SPEER, M.D. M.R.I.A. late Physician to the Dublin General Dispensary. —pp. 132. Cambridge, 1823.

Case of Transverse Fracture of the Patella, in which perfect Osseous Union was produced; with Observations. By GEORGE FIELDING, Mem. Royal College of Surgeons, Edinburgh; one of the Surgeons to the Infirmary, to the Lying-in Charity, &c. &c.—Hull, 1823.

Lettere del Professore VACCA BERLINGHIERI al Professore ANTONIO SCARPA, sulla Ligatura delle grosse Arterie e degli Arti. pp. 32.—Pisa.

Archiv für Medizinische Erfahrung im Gebiete der Praktischen Medizin Chirurgie, Geburtshülfe, und Staatsarzneikunde.—Berlin.

Leçons sur les Epidemies et l'Hygiène publique faites a la Faculté de Medecine de Strasbourg. Par F. E. FODERÉ, Professeur a cette Faculté. Tome premier. pp. 523. Paris, 1822.

---

### NOTICES TO CORRESPONDENTS.

*The following* PAPERS *are preparing for the Press:*—Mr. SHAW *on the Respiratory Nerves* (concluded); Dr. FORBES *on Inflammation of the Mucous Membrane of the Lungs;* Mr. MARSHALL *on the Health of the Troops in Scotland;* Mr. JEFFERY's *Case of imperforate Duodenum;* Dr. R. HARRISON *on the Ligature of Arteries;* Dr. KINGLAKE *on the Medicinal Powers of Elaterium, and on Renal Inflammation; and* Dr. MACLEOD *on the Diseases prevalent in London.*

*We have received* Mr. FOSBROKE's *Communication. We beg to refer him to our last Number, in which we have already given publicity to the intention of erecting a monument to* Dr. JENNER, *at Gloucester.*

*We regret that we cannot give insertion to* Mr. YEATMAN's *paper. We believe our readers have already had quite enough of the controversy between* Mr. BUSH *and him. It may be proper to state, that Mr. Y. affirms that the fractured ends of the bone, in the case of Wolf, united without any displacement.*

*We have received Reports of the Eye Institutions at Plymouth and Newcastle: we regret that the interest they possess is of too local a nature for insertion in this Journal.*

